# Tantalum Oxide as an Efficient Alternative Electron Transporting Layer for Perovskite Solar Cells

**DOI:** 10.3390/nano12050780

**Published:** 2022-02-25

**Authors:** Meenal Deo, Alexander Möllmann, Jinane Haddad, Feray Ünlü, Ashish Kulkarni, Maning Liu, Yasuhiro Tachibana, Daniel Stadler, Aman Bhardwaj, Tim Ludwig, Thomas Kirchartz, Sanjay Mathur

**Affiliations:** 1 Institute of Inorganic Chemistry, University of Cologne, Greinstr. 6, 50939 Cologne, Germany; meenald@srmist.edu.in (M.D.); amoellmann@gmx.de (A.M.); feray.uenlue@uni-koeln.de (F.Ü.); daniel.stadler@nmwp.de (D.S.); aman.bhardwaj@uni-koeln.de (A.B.); tim.ludwig88@gmx.de (T.L.); 2 Department of Physics and Nanotechnology, SRM Institute of Science and Technology, Kattankulathur, Chennai 603203, India; 3 IEK5-Photovoltaics, Forschungszentrum Jülich, 52425 Jülich, Germany; j.haddad@fz-juelich.de (J.H.); a.kulkarni@fz-juelich.de (A.K.); t.kirchartz@fz-juelich.de (T.K.); 4 School of Engineering, RMIT University, Bundoora, VIC 3083, Australia; maning.liu@tuni.fi (M.L.); yasuhiro.tachibana@rmit.edu.au (Y.T.)

**Keywords:** electron transport material, amorphous Ta_2_O_5_, n-type semiconductor, perovskite solar cell

## Abstract

Electron transporting layers facilitating electron extraction and suppressing hole recombination at the cathode are crucial components in any thin-film solar cell geometry, including that of metal–halide perovskite solar cells. Amorphous tantalum oxide (Ta_2_O_5_) deposited by spin coating was explored as an electron transport material for perovskite solar cells, achieving power conversion efficiency (PCE) up to ~14%. Ultraviolet photoelectron spectroscopy (UPS) measurements revealed that the extraction of photogenerated electrons is facilitated due to proper alignment of bandgap energies. Steady-state photoluminescence spectroscopy (PL) verified efficient charge transport from perovskite absorber film to thin Ta_2_O_5_ layer. Our findings suggest that tantalum oxide as an n-type semiconductor with a calculated carrier density of ~7 × 10^18^/cm^3^ in amorphous Ta_2_O_5_ films, is a potentially competitive candidate for an electron transport material in perovskite solar cells.

## 1. Introduction

The emergence of organic-inorganic hybrid perovskite solar cells (PSCs) and the coherent efforts of researchers across the world have led to impressive progress in power conversion efficiency (PCE) from merely 3.8% to 25.5% within a short span of time [1,2,3,4,5]. This vertical growth is mostly attributed to the facile processing chemistry and exceptional optoelectronic properties of hybrid organic-inorganic perovskites (e.g., (Me_2_N)PbI_3_), such as their suitable bandgaps, high optical absorption coefficients, long carrier diffusion lengths and high carrier mobility [6,7]. Given their high PCEs and expanding market presence, PSCs are the optimal choice for the envisaged integrated self-charging power packs [8]. In a typical thin-film solar cell structure, the perovskite absorber layer is sandwiched between an electron transporting layer (ETL) and a hole transporting layer (HTL) [9,10]. Among the class of ETLs, TiO_2_ has been extensively studied together with a few other broad bandgap semiconductors (SnO_2_, ZnO and Zn_2_SnO_4_) applied in both pristine and doped compositions as mesoporous or compact layers [11,12,13]. Photovoltaic cells with PCE beyond 20% have been fabricated mostly using TiO_2_ ETL; however, they have some intrinsic limitations, including misalignment between the conduction band of TiO_2_ and the currently most studied triple-cation-based perovskite material, which leads to strong hysteresis that can be observed during the current (J)–voltage (V) measurements [14]. In addition to this, the UV-sensitive TiO_2_ not only generates trap and defect centers, but also degrades the perovskite layer [15,16,17]. Therefore, it is essential to explore alternative ETLs that combine high photostability and good electronic compatibility among functional components (ETL, HTL, photo absorber) with low defect densities at the ETL/perovskite interface. In this context, tantalum oxide is one of the yet unexplored but potentially promising materials owing to its high transparency, chemical stability and favorable band alignment with perovskite, as demonstrated in this study for a triple-cation mixed-halide perovskite (Cs_0.05_(MA_0.17_FA_0.83_)_(0.95)_Pb(I_0.83_Br_0.17_)_3_).

Tantalum oxide (Ta_2_O_5_) is an important dielectric material for electronic devices due to its high dielectric constant (~25) [18]. Although the bandgap of Ta_2_O_5_ is reasonably wide (~3.9 eV), it has recently been explored for photocatalytic water splitting applications in composite form [19,20,21,22,23,24] and various nanostructures [25,26,27,28,29,30,31,32]. Owing to its large bandgap energy, Ta_2_O_5_ has been widely used as an anti-reflection coating—for example, on silicon solar cells [33,34,35] to increase light absorption and thus overall current density of the solar cell. Tantalum oxide nanowire [36] and composites of tantalum oxide with P25 [37] have also been successfully used as photoanode materials in dye-sensitized solar cells (DSSCs) and as electron-selective contact layers in indium phosphide (InP) solar cells [38]. Ta_2_O_5_-ZnO composite film has been used as a cathodic buffer layer for inverted polymer solar cells to get enhanced efficiency [39]. In an interesting work by Ying et al., a combination of ZrAcac/PEI/Ag/Ta_2_O_5_ was employed as the rear transparent electrode of semi-transparent PSCs, leading to enhanced transmittance [40]. It is suggested that materials with high dielectric constants could offer better charge separation and improve the photovoltaic efficiency by suppressing the carrier recombination across the interfaces [41,42,43]. This suggests that Ta_2_O_5_ will offer strong electronic charge separation and fewer defect sites for charge recombination. Additionally, the favorable band alignment would ensure efficient electron transport across the interfaces, which would make amorphous Ta_2_O_5_ a potential ETL for PSCs. Considering these factors, Liu and co-workers speculated on the potential of Ta_2_O_5_ as an ETL material in halide perovskite-based solar cells [44]. Chavan et al. [45,46] employed Ta_2_O_5_ as a passivation layer between mesoporous TiO_2_ and perovskite layers and observed an increase in open circuit voltage, leading to better performances. Despite these efforts, Ta_2_O_5_ has not been explored in all dimensions, emphasizing both the potential and the necessity for further investigation in this field.

Although the recent work by Chavan et al. [46] has highlighted the importance of Ta_2_O_5_ as a passivation layer in TiO_2_-based perovskite solar cells, they deposited the Ta_2_O_5_ layer by atomic layer deposition (ALD). ALD offers controlled growth of the film, but it is an expensive technique compared to the solution processable methods. In the present work, we employed a simple spin coating method to deposit the tantalum oxide layer. We successfully replaced the widely used TiO_2_ compact ETL with spin coated amorphous Ta_2_O_5_ in perovskite solar cells. Amorphous Ta_2_O_5_ layers were deposited on fluorine doped tin oxide (FTO)-coated glass via spin coating a tantalum ethoxide precursor solution (various concentrations), followed by annealing in air at 500 °C. With optimized Ta_2_O_5_ compact film (10 nm) as the ETL, the perovskite device showed a maximum PCE of 14%. Ultraviolet photoelectron spectroscopy (UPS) measurements revealed suitable band alignment of Ta_2_O_5_ with that of perovskite, leading to better charge transport. This was further corroborated by steady-state photoluminescence (PL) measurements demonstrating efficient electron injection from perovskite to the thin Ta_2_O_5_ layer.

## 2. Experimental

### 2.1. Deposition of Ta*_2_*O*_5_* Films and Fabrication of Perovskite Solar Cells

**Scheme 1:**
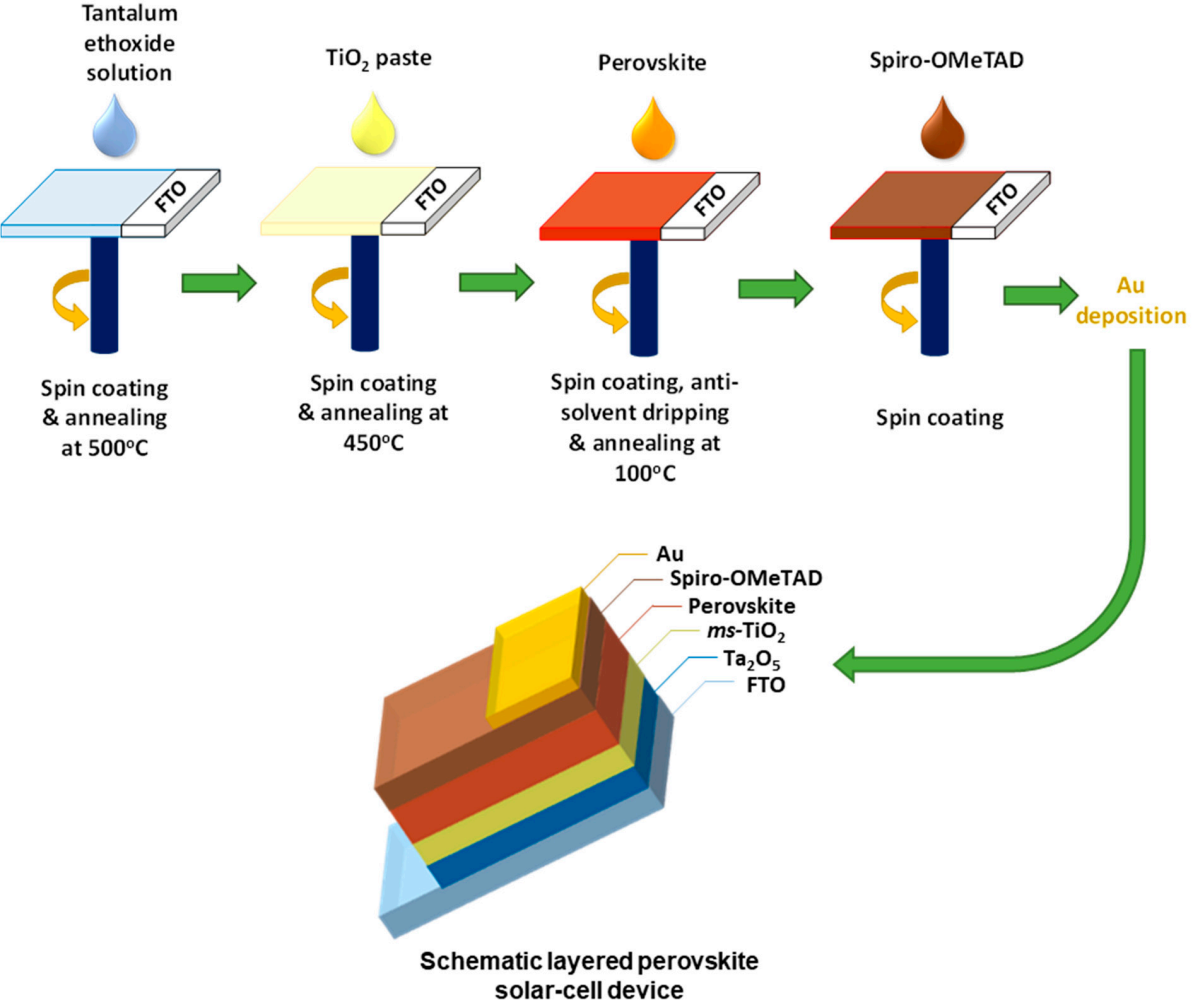
Schematic diagram of the procedure for preparation of perovskite solar cells and the resulting layered PSC device.

Tantalum (V) ethoxide (99.98%) was purchased from Sigma-Aldrich (St. Louis, MO, USA) and used for the solution preparation without further purification. The tantalum oxide layers were fabricated using tantalum (V) ethoxide solutions in ethanol that were diluted to achieve the desired solution concentrations (0.02, 0.05, 0.1 and 0.2 M) inside a dry nitrogen glovebox. These tantalum ethoxide precursor solutions were spin-coated on the cleaned FTO substrates at 1000 rpm with 200 rpm/s acceleration for 30 s following and in a second step at 3000 rpm with 1500 rpm/s acceleration for 30 s. Meanwhile, the FTO contact area was covered with Kapton tape. The thin films were heated to 100 °C for 10 min prior to removing the Kapton tape, followed by annealing at 500 °C for 1 h in air. After the deposition of tantalum oxide films, a step-by-step perovskite solar cell fabrication procedure was followed—the one reported in our earlier work [47] and shown in Scheme 1. The detailed device fabrication and characterization procedure can also be found in Section S1 in the Supporting Information.

### 2.2. Characterization of Deposited Ta*_2_*O*_5_*

Scanning electron microscopic (SEM) images were taken using a NEON40 (ZEISS, Jena, Germany) equipped with a Gemini-gun (InLens and secondary electron detector). The samples were sputtered with gold and touched with copper tape to prevent charging effects. The imaging and analysis of the surface morphology by atomic force microscopy (AFM) was measured with an XE-100 (Park Systems, Suwon, Korea) with a cantilever of the type PPP-NHCR-10 (Nanosensors, Neuchatel, Switzerland). The measurements were taken in a non-contact mode using a scan size of 5 µm × 5 µm and a speed of 1 Hz yielding images with 256 pixel × 256 pixel resolution and a tip radius of curvature <10 nm. The measurements were done under ambient conditions in air. The surface roughness (*R*_q_) was determined by XEI 1.8.0 software. A NanoCalc-XR (Ocean Optics, Ostfildern, Germany) refractometer was used to measure a reflection spectrum of the amorphous Ta_2_O_5_ layer on the silicon wafer and calculate the film thicknesses. The technique is based on the well-known interference of light with thin films. The X-ray diffractogram was measured in reflection geometry with a STOE-STADI MP device (Darmstadt, Germany) with a linear PSD detector using a Mo-Kα X-ray (λ = 0.71 nm) source. X-ray photoelectron spectroscopy (XPS) analysis was performed with an ESCA M-Probe (Surface Science Instruments) employing Al-Kα X-rays (1486.6 eV). No sputtering of the sample was performed prior to the measurements. Adventitious carbon (C 1s) at 284.8 eV was used as reference for the binding energy and the peaks were fitted using CasaXPS software. The transmittance was measured with a Lambda 950 spectrometer (Perkin-Elmer, Waltham, MA, USA). Mott–Schottky measurements were measured in a three-electrode set-up with an Agilent E49801 Precision LCR Meter (Santa Clara, CA, USA) for measuring the capacitance and a 2400 series SourceMeter (Keithley instruments, Cleveland, OH, USA) for sweeping the potential. Platinum was used as a counter electrode, a saturated calomel electrode as reference electrode and coated FTO substrates as a working electrode. Ultraviolet photoelectron spectroscopy (UPS) was performed in an ultra-high vacuum (UHV) on an EAC2000 SPHERA 547 spectrometer (Omicron Nanotechnology ESCA, Taunusstein, Germany) with an ARGUS Energy Analyser. He I discharge lamp (21.2 eV) was used for excitation with an energy resolution of <105 mV under a bias voltage of −9.0 V. The spectra were calibrated against the gold Fermi level to determine the work function and against vacuum level for the estimation of the valence band using the cut-off energy.

UV–Vis absorption spectra of the glass/perovskite, glass/c-TiO_2_/perovskite and glass/Ta_2_O_5_/perovskite films were taken in a spectrometer (Shimadzu, UV-2450, Kyoto, Japan). Steady-state photoluminescence spectra of the thin films were measured between 400 and 800 nm using a PTI UV-Vis fluorometer (Photon Technology International, Inc., Birmingham, NJ, USA). A slit width of 1.0 mm (4.0 nm resolution) was used at room temperature. A photomultiplier was used for detection and corrected for the spectral response of a grating in the emission monochromator and the detector. Transient photoluminescence decay profiles of the thin films were obtained by a home-built transient emission spectrometer equipped with an N_2_ laser (LTB Lasertechnik Berlin GmbH, MNL 202-C, Berlin, Germany) pumped dye laser (LTB Lasertechnik Berlin GmbH, ATM200, 700 ps pulse duration) as an excitation source, a monochromator (Princeton Instruments, Acton, MA, USA), a nanosecond detection system (Unisoku Co., Ltd., Osaka, Japan, TSP-2000SN, with 1.2 ns time resolution (FWHM)) and a fast oscilloscope (Tektronix, Beaverton, OR, USA, TDS 3052C, Digital Phosphor Oscilloscope 500 MHz 5 GS/s) at 10 Hz excitation repetition rate. The emission decay profile was synchronized with the excitation pulse and the detection system using a laser trigger detector.

## 3. Results and Discussion

For simplicity, solar cells with spin-coated Ta_2_O_5_ layers on FTO substrates via various concentrations of tantalum ethoxide solutions are denoted as Ta1 (0.02 M), Ta2 (0.05 M), Ta3 (0.10 M) and Ta4 (0.20 M). Moreover, a reference device was fabricated without any blocking layer (Ta0) to show the beneficial effect of adding tantalum oxide to an ETL.

**Figure 1:**
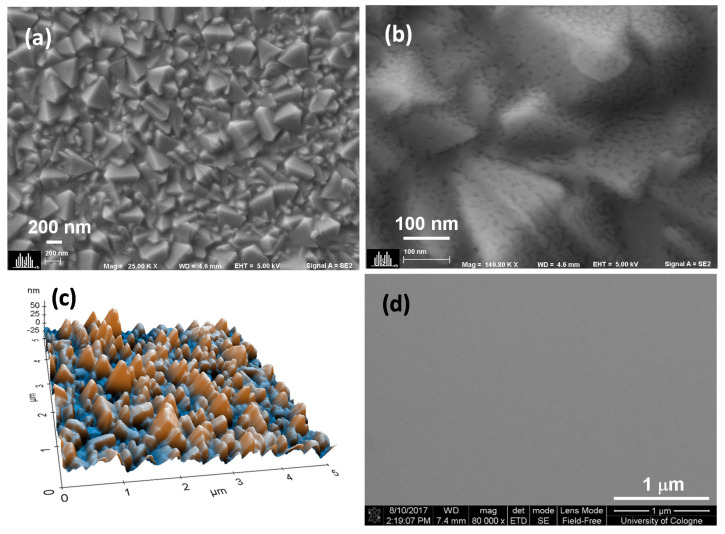
Morphology of Ta1 using scanning electron microscopy (SEM) at (**a**) low and (**b**) high magnifications, (**c**) 3D topography using AFM and (**d**) SEM of the Ta_2_O_5_ film on the silicon substrate.

Figure 1a shows a top view scanning electron microscope (SEM) image of the film deposited using a 0.02 M solution (Ta1) on FTO. The low magnification SEM image revealed similar topography that compared well with the morphology of bare FTO (Figure S1a) however, a uniform film formation and seamless coverage on the FTO surface were visible at higher resolution (Figure 1b). These findings were similar for all tantalum ethoxide concentrations used in this work (Figure S2). The 3D topography using non-contact atomic force microscopy (NC-AFM), as shown in Figure 1c, showed a highly rough surface (16 nm). The roughness is mainly attributed to the FTO substrate exhibiting a roughness of 18 nm (Figure S1b). Further AFM images corresponding to all other Ta_2_O_5_ containing films (Ta2–Ta4) are shown in Figure S1, and the root mean square roughness (*R*_q_) values obtained from AFM are listed in Table 1. The roughness values of Ta1 and Ta2 samples are comparable to that of the bare FTO. It was observed that the roughness gradually reduced upon increasing the concentration (i.e., for Ta3 and Ta4) of the tantalum ethoxide solution, which indicated increased coverage of FTO surface voids. To evaluate the uniformity of the fabricated layer without the influence of the underlying FTO structure, the tantalum oxide films were deposited on silicon substrates. The films were found to be highly compact, without any pinholes or agglomerates, as shown in Figure 1d and Figure S3a. The reference TiO_2_ film deposited by spray pyrolysis displayed the formation of particulate agglomerates (Figure S3b), in contrast to the homogenous and smooth spin-coated tantalum oxide films. We used to determine the film thickness by optical reflectometry. Table 1 shows an increase in film thickness from 9 nm (Ta1) to 19 nm (Ta4). A linear increase in Ta_2_O_5_ film thickness with an increasing solution concentration of the tantalum ethoxide was expected from a spin coating process and was evident in the measured roughness values (Table 1).

**Figure 2:**
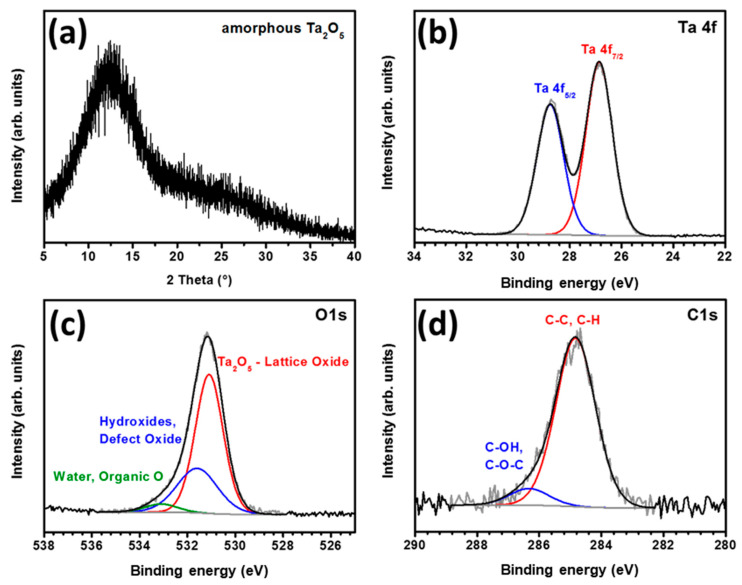
(**a**) XRD pattern of tantalum oxide film deposited on glass; XPS spectra of tantalum oxide showing high-resolution peaks corresponding to (**b**) Ta 4f, (**c**) O 1s and (**d**) C 1s.

To evaluate the crystallinity and phase of the tantalum oxide film, X-ray diffraction (XRD) of Ta4—viz., the sample with the highest thickness, deposited using 0.2 M tantalum ethoxide solution on bare glass followed by annealing at 500 °C for 1 h in air—was recorded. The XRD (Figure 2a) confirmed the amorphous nature of the film. Interestingly, the thermogravimetric analysis (TGA) of tantalum ethoxide, measured in air and reported in the literature, shows major mass loss with the evolution of CO_2_ and H_2_O, leading to conversion of the gel to tantalum oxide at a lower temperature (~305 °C) [48] However, Ta_2_O_5_ crystallizes at temperatures above 650 °C, which is impractical for solar cell fabrication on FTO substrates. Hence, a rather moderate temperature of 500 °C was chosen in this work to ensure the formation of a stoichiometric oxide necessary for the subsequent fabrication of perovskite solar cells.

The electronic states and compositions of the tantalum oxide films were determined using X-ray photoelectron spectroscopy (XPS). The XPS survey spectrum is shown in Figure S4. As seen in Figure 2b, two peaks observed at 28.7 and 26.8 eV were assignable to 4f_5/2_ and 4f_7/2_, respectively, corresponding to the Ta^+5^ oxidation state, which confirmed the formation of stoichiometric Ta_2_O_5_ after annealing of the film, since the Ta^+5^ component was the only phase detected in Ta 4f region [49]. The O 1s region (Figure 2c) exhibited the main peak at 531.1 eV attributed to the lattice oxide, confirming the presence of an O^2−^ state in Ta_2_O_5_. The higher binding energy peak centered in the region 531.6–533.1 eV can be assigned to chemisorbed oxygen, defect oxygen atoms or hydroxyl groups and possibly to surface-adsorbed water molecules or organic oxygen (residual ethoxide groups) [49]. The peaks in the C1s spectrum at 284.8 and 286.4 eV in Figure 2d correspond to C–C, C–H and C–OH or C–O–C components, respectively. This spectrum matches well with the presence of adventitious carbon indicating the adsorption of impurities on the film surface after exposing it to air.

The flat band potential and carrier density of the Ta_2_O_5_ film were derived from capacitance–voltage (C–V) characteristics in the electrochemical setup to obtain the Mott–Schottky plot, in which 1/*C*^2^ is plotted against the applied potential. The Mott–Schottky Equation (1) is given as

(1) $$\frac{1}{C^{2}}=\frac{2}{e\varepsilon \varepsilon_{0}A^{2}N_{d}}\;\left(V-V_{\mathrm{fb}}-\frac{k_{B}T}{e}\right)$$

where *ε*_0_ is the permittivity of free space; *ε* is the relative permittivity of amorphous Ta_2_O_5_, which is ~20 [12]; *e* is the electronic charge; *N_d_* is the charge carrier density; *A* is the area of the film in contact with the electrolyte; and *V*_fb_ is the flat band potential. We can get the value of the flat band potential and the charge density through the intercept of linear portion of 1/*C*^2^ on the voltage axis as:

(2) $$N_{d}=\frac{2}{e\varepsilon \varepsilon_{0}A^{2}}\;{\left[\frac{d\left(\frac{1}{C^{2}}\right)}{dV}\right]}^{-1}$$

The value of flat band potential obtained from Mott–Schottky plot shown in Figure S5 is −0.8 V vs. NHE. The positive slope of the Mott–Schottky plot confirmed the amorphous Ta_2_O_5_ to be an n-type semiconductor with a calculated carrier density of ~7 × 10^18^/cm^3^ in the amorphous Ta_2_O_5_ films deposited on FTO. In comparison, from Mott–Schottky measurements of compact TiO_2_ thin films, a carrier density of ~8.3 × 10^16^/cm^3^ was reported [50].

**Figure 3:**
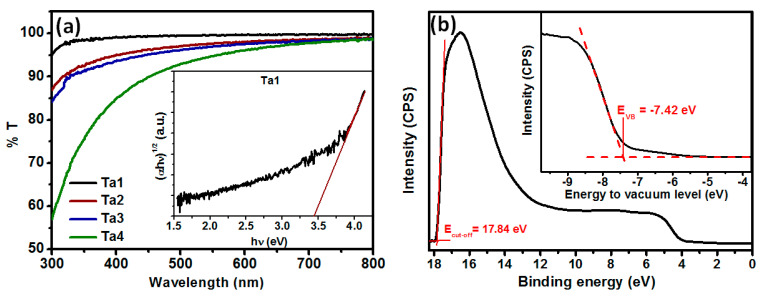
(**a**) Transmission spectra measured by UV–Vis spectrometry of Ta_2_O_5_ films of thicknesses ~9 to ~19 nm; inset shows Tauc plot of Ta1 film. (**b**) UV photoelectron spectroscopy of Ta_2_O_5_ film; inset shows the UPS spectrum in the valence band region.

The high optical transmission of the ETL is essential in order to enable maximum light availability for perovskite absorption. Figure 3a shows the transmission spectra of Ta_2_O_5_ films deposited on FTO by using various concentrations of tantalum ethoxide. With increasing precursor concentration there was a concomitant increase in film thickness (Table 1), which reduced the transmission of the film in the visible region.

Ta1 was found to be highly transparent. Transmission reached ~99% at 450 nm. Transmission was ~90% for Ta4 due to increased film thickness. The decrease in %T with increasing thickness can be assigned to the increasing carbon content resulting from the unreacted precursor, as tantalum ethoxide does not get completely decomposed at 500 °C, as reported earlier [48]. The inset of Figure 3a shows the Tauc plot for Ta1 that was used to calculate the optical bandgap of the amorphous Ta_2_O_5_ film (~3.45 eV), which compares well with the values reported earlier for Ta_2_O_5_ films annealed at 500 °C [26]. To estimate the exact positions of electronic bands in our amorphous Ta_2_O_5_, we have performed UPS measurements (Figure 3b) for Ta1 film deposited on FTO. The inset shows a magnified valence band region, in which the valence band maximum (VBM) or E_VB_ position is extrapolated. The VBM was determined to be −7.42 eV below vacuum level. The determined valence band and measured spectra of our amorphous Ta_2_O_5_ are in good agreement with values reported for crystalline Ta_2_O_5_ [51].

**Figure 4:**
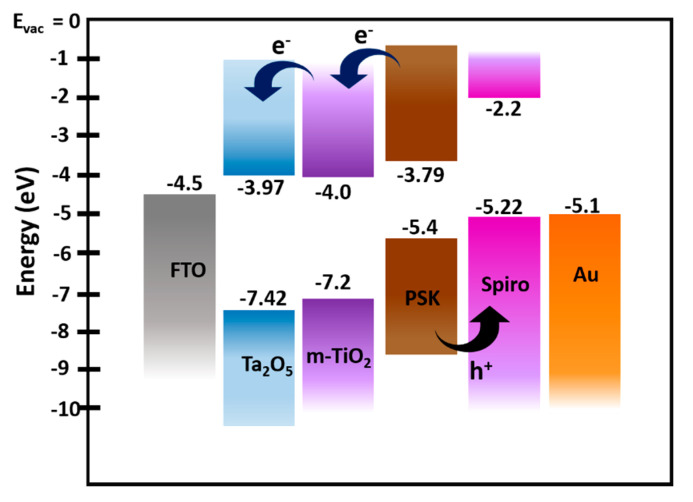
Energy level diagram of FTO/amorphous Ta_2_O_5_/meso-TiO_2_/perovskite/spiro-OMeTAD layered stacks. The energy level for Ta_2_O_5_ was determined by UPS and UV–Vis spectroscopy, and for other device stack layers, the values were adopted from the literature.

The energy level diagram of the perovskite solar cell architecture used in this study is given in Figure 4. Considering the VBM of Ta_2_O_5_ at −7.42 eV and the bandgap of ~3.45 eV, the conduction band minimum was estimated to be at −3.97 eV. The values for perovskite and energy levels of other components were taken from the literature [52]. This favorable band alignment of the materials used in the devices ensures efficient electron transport from the perovskite layer to the amorphous Ta_2_O_5_, as observed in our devices, which makes amorphous Ta_2_O_5_ a potential ETL for PSCs.

To confirm that the conduction band edge potential of Ta_2_O_5_ is more negative compared to that of a TiO_2_ compact layer, which is favorable for efficient electron injection from the perovskite conduction band to that of the ETL, photoluminescence (PL) quenching measurements were conducted by replicating the stack layers on thin glass substrates and employing glass/c-TiO_2_/perovskite, glass/Ta1/perovskite (thinnest Ta_2_O_5_) and glass/perovskite configurations. Figure S6 shows absorption spectra of the above perovskite thin films. The absorption amplitude was similar at all wavelengths, suggesting that the optical thickness of the perovskite layer was similar in all films. Steady-state PL spectra (Figure S7) and transient PL (TRPL) decays (Figure S8) were measured for the above perovskite films. Since glass is an insulator, no charge transfer process was expected, and accordingly, the glass/perovskite sample showed strong photoluminescence and slow quenching. The PL amplitudes of both c-TiO_2_/perovskite and Ta1/perovskite were reduced, and TRPL measurements showed a slightly faster decrease in PL compared to the glass/perovskite reference. This suggests that the electron injection occurs from the perovskite conduction band to the compact ETL (TiO_2_ or Ta_2_O_5_) conduction band, followed by charge accumulation and non-radiative recombination at the ETL/perovskite interface [53].

**Figure 5:**
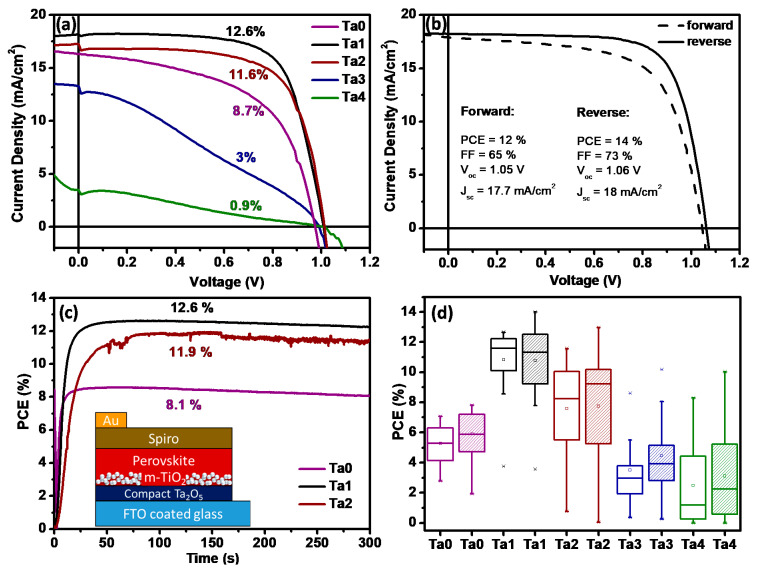
Comparison of (**a**) J–V curves (forward sweep). (**b**) Champion cell of perovskite solar cells employing Ta_2_O_5_ as ETL (Ta1). (**c**) Steady-state power conversion efficiency at maximum power point (0.78 V applied bias); inset shows a schematic of the layered stack used for the devices. (**d**) Box plots displaying the distribution of device efficiencies fabricated on Ta0, Ta1, Ta2, Ta3 and Ta4 films (forward and reverse sweep).

The J–V curves (forward-biased) recorded for the typical devices are displayed in Figure 5a to compare the influence of the addition of Ta_2_O_5_ layer on the photovoltaic performance of the devices. Figure 5a shows the PCE increment from 8.7% for Ta0 to 12.6% through addition of a thin tantalum oxide blocking layer (Ta1). To further analyze the photovoltaic performance of the new solar cells employing Ta_2_O_5_-based ETLs, fill factor (FF) was calculated (the ratio of maximum obtainable power to the product of the open-circuit voltage and short-circuit current). The standard method for the determination of fill factor for Ta_2_O_5_-ETL solar cells operated in reverse bias is illustrated in Figure S9 in the Supplementary Information. Devices without a blocking layer exhibited a fill factor of 47%, a short-circuit current density (J_sc_) of 14.4 mA/cm^2^ and an open-circuit voltage (V_oc_) of 0.92 V. Employing Ta_2_O_5_ as the ETL substantially increased overall efficiency by an enhanced FF of 65%, J_sc_ of 16.4 mA/cm^2^ and V_oc_ of 0.99 V. Figure 5b shows J–V curves of the Ta1 champion device, which exhibited V_oc_ exceeding 1 V, FF ≈ 73% and a PCE of 14% in a reverse sweep. The significant improvement in FF substantiates the improved PV properties of the solar cell employing Ta_2_O_5_ as an ETL. The forward and reverse biased curves also reveal low hysteresis behavior. Although the overall photovoltaic performance of the devices was not very good compared to the performance of the perovskite solar cells reported by other groups recently, the efficiency after employing Ta_2_O_5_ as ETL was quite good compared to our reference device, as shown in Figure S10. For our reference devices using standard compact and mesoporous TiO_2_ layers, we obtained an average PCE of ~10%. The averaged photovoltaic parameters, such as PCE, FF, V_oc_, J_sc_ and R_s_, which were extracted from the J–V curves and the number of measured cells, are summarized in Table 2.

A distribution of PCE is summarized in Figure 5d as a box plot diagram. Comparing the averaged photovoltaic parameters using Ta_2_O_5_, we can conclude that the incorporation of the new ETL is beneficial for the overall performance enhancement in perovskite devices. The advantageous effect reached a threshold value at the Ta_2_O_5_ layer thickness of 12 nm (Ta2), after which the dielectric properties of the tantalum oxide blocking layer inhibited electron transport due to increasing series resistance that hiked from 8 Ω cm^2^ for Ta1 and Ta2 to 119 Ω cm^2^ for the 19 nm Ta_2_O_5_ layer. The high series resistance resulted in a decreased FF of 55% and a J_sc_ of 7.1 mA/cm^2^. Maximum power point (MPP) tracking measurements (Figure 5c) were conducted to show the device efficiency of Ta0, Ta1 and Ta2 devices under steady-state conditions. The MPP conversion efficiencies were calculated based on the chronoamperometry measurement over 300 s (Figure S11). Estimated power conversion efficiencies of 12.6% (Ta1), 11.9% (Ta2) and 8.1% (Ta0) match precisely to the efficiencies extracted from the J–V curves. MPP tracking plots for Ta3 and Ta4 solar cells are given in Figure S12, which show an expectedly inferior performance.

External quantum efficiencies (EQE) were measured to evaluate the effect of a tantalum oxide blocking layer on the photon-to-current conversion efficiency depending on the wavelength (Figure S13). The integrated photocurrent density values have also been calculated from EQE. The general trend of decreasing photocurrent density with increasing tantalum oxide layer thickness was also confirmed by EQE measurements. The exemplary Ta1 device showed an integrated current density of 15.8 mA/cm^2^; juxtapose that with 10.7 mA/cm^2^ for a Ta4 solar cell. Besides the overall decrease in current density, the Ta4 solar cell displayed atypical curve progression in the region between 350 and 500 nm in comparison to the other devices. Light absorption or weak charge transfer of the thick Ta4 blocking layer in this wavelength range could prevent efficient photon-to-current conversion.

The distribution of sample efficiency is reasonable and can be explained by general deviations in sample preparation. Although the distribution is imperfect, the general improvement caused by the addition of thin tantalum oxide layers is unambiguously evident. Details on the scattering of photovoltaic parameters, such as V_oc_, J_sc_ and FF, are given in the box plots in Figure S14. From the abovementioned results, we can see that unexplored amorphous tantalum oxide can function as an electron transport material (ETM) in the perovskite solar cell. Although the efficiency is not high as compared to the traditional TiO_2_-based perovskite devices, which usually show efficiency ≥20%, we believe that further optimization can help to improve the performance of the tantalum oxide-based perovskite devices. The high performance of TiO_2_-based devices is not always compatible with stability, mainly because of the UV-sensitive nature of the TiO_2_ compact layer. Researchers have also shown that interface modification of TiO_2_ compact layer and/or completely replacing the TiO_2_ compact layer, and even compact-layer-free perovskite devices, deliver similar V_OC_ and have improved stability [16,54,55]. Thus, exploring amorphous tantalum oxide as an ETM is just a small step toward replacing TiO_2_ in order to achieve stable perovskite solar cells.

## 4. Conclusions

We have demonstrated the incorporation of thin amorphous Ta_2_O_5_ interfacial layers as alternative ETLs in perovskite solar cells to replace the commonly used TiO_2_ blocking layer. We evaluated the beneficial influence on photovoltaic performance, achieving champion solar cell performance of ~14%. We have explained the improvement in device performance by favorable valence band alignment of Ta_2_O_5_, as determined by UPS and Tauc plots. The Ta_2_O_5_ thickness was found to be crucial for the series resistance, short-circuit current and PCE because of the highly dielectric behavior of tantalum oxide. This could prevent efficient current flow after a certain film thickness threshold. Steady-state PL measurements also indicate efficient electron injection from excited perovskite to the Ta_2_O_5_ layer. As per our knowledge, this is the first report of the employment of amorphous Ta_2_O_5_ in PSCs, and there are several opportunities for improving the tantalum oxide film’s properties—e.g., by doping or by depositing Ta_2_O_5_ via ALD for a conformal coating on FTO. Therefore, it is likely that fabrication optimization will result in highly efficient solar cells that could inhibit photo-corrosion, which is a persisting challenge for devices exposed to long-term solar illumination.

## Figures and Tables

**Table 1:** Roughness and thickness values of tantalum oxide films deposited on FTO and silicon, respectively.

Sample Name	Precursor Concentration	Roughness (*R*_q_, from AFM)	Average Thickness (nm)
**Ta0**	0 M	18 nm	-
**Ta1**	0.02 M	16 nm	9
**Ta2**	0.05 M	16 nm	12
**Ta3**	0.10 M	14 nm	15
**Ta4**	0.20 M	11 nm	19

**Table 2:** Photovoltaic parameters of PSCs, using amorphous Ta_2_O_5_ layers of different thicknesses (Ta0–Ta4) as their ETLs.

Sample	No. of Cells	PCE (%)	FF (%)	V_oc_ (V)	J_sc_ (mA/cm^2^)	Rs (Ω cm^2^)
**Ta0**	12	6.4	47	0.91	14.4	6
**Ta1**	18	10.8	66	0.99	16.5	8
**Ta2**	26	8.9	61	0.94	15.6	8
**Ta3**	24	3.0	36	0.87	10.2	23
**Ta4**	20	2.8	55	0.79	7.1	119

## Data Availability

The data presented in this study are available on request from the corresponding author.

## Supplementary Materials

The following are available online at https://www.mdpi.com/article/10.3390/nano12050780/s1, Figure S1: (a) Scanning electron microscopy (SEM) and (b) Atomic force microscopy (AFM) and (d) scanning electron microscopy images of bare FTO, i.e., Ta0; AFM of the films (c) (b) Ta1, (dc) Ta2, (e) Ta3 and (f) Ta4, Figure S2: Morphologies of the films Ta1, Ta2, Ta3 and Ta4 by SEM with low and high magnifications, Figure S3: Surface morphologies of the films: (a) Ta_2_O_5_ by spin coating and (b) TiO_2_ by spray pyrolysis prepared on silicon substrate, Figure S4: XPS survey spectrum of tantalum oxide thin film, Figure S5: Mott–Schottky plot of Ta3 film dipped in 0.1 M Na_2_SO_4_ as an electrolyte recorded at 1 kHz, Figure S6: Absorption spectra of glass/perovskite, glass/c-TiO_2_/perovskite and glass/Ta_2_O_5_/perovskite with ~9 nm Ta_2_O_5_ layer thickness (Ta1), Figure S7: Normalized steady state photoluminescence spectra of glass/perovskite, glass/c-TiO_2_/perovskite and glass/Ta_2_O_5_/perovskite with ~9 nm Ta_2_O_5_ layer (Ta1) at 625 nm excitation, Figure S8: Transient photoluminescence spectra of glass/perovskite, glass/c-TiO_2_/perovskite and glass/Ta_2_O_5_/perovskite with ~9 nm Ta_2_O_5_ layer (Ta1) at 625 nm excitation, Figure S9: Illustration of the determination of fill factor for Ta1 champion device in reverse bias, Figure S10: Reference cell J–V curves of perovskite solar cells employing compact and mesoporous TiO_2_ as ETL, Figure S11: Measured photocurrent density of PSCs at maximum power (0.78 V applied bias) with varying tantalum oxide thickness, Figure S12: Calculated device efficiency of Ta3 and Ta4 PSCs from maximum power (0.78 V applied bias) tracking over 300 s, Figure S13: External quantum efficiency (EQE) spectra and corresponding integrated photocurrent densities of typical perovskite solar cells varying in Ta_2_O_5_ layer thickness, Figure S14: Box plot diagrams for the distribution of FF, V_oc_, J_sc_ and R_s_ (only reverse sweep) extracted from the J–V curves. References [56,57,58] are cited in the supplementary materials.

## References

[1] NREL Best Research-Cell Efficiency Chart. https://www.nrel.gov/pv/cell-efficiency.html.

[2] Kojima A., Teshima K., Shirai Y., Miyasaka T. (2009). Organometal Halide Perovskites as Visible-Light Sensitizers for Photovoltaic Cells. J. Am. Chem. Soc..

[3] Jena A.K., Kulkarni A., Miyasaka T. (2019). Halide Perovskite Photovoltaics: Background, Status, and Future Prospects. Chem. Rev..

[4] Yoo J.J., Seo G., Chua M.R., Park T.G., Lu Y., Rotermund F., Kim Y.-K., Moon C.S., Jeon N.J., Correa-Baena J.-P. (2021). Efficient perovskite solar cells via improved carrier management. Nature.

[5] Min H., Lee D.Y., Kim J., Kim G., Lee K.S., Kim J., Paik M.J., Kim Y.K., Kim K.S., Kim M.G. (2021). Perovskite solar cells with atomically coherent interlayers on SnO_2_ electrodes. Nature.

[6] De Quilettes D.W., Koch S., Burke S., Paranji R.K., Shropshire A.J., Ziffer M.E., Ginger D.S. (2016). Photoluminescence Lifetimes Exceeding 8 Μs and Quantum Yields Exceeding 30% in Hybrid Perovskite Thin Films by Ligand Passivation. ACS Energy Lett..

[7] Brenner T.M., Egger D.A., Kronik L., Hodes G., Cahen D. (2016). Hybrid Organic—Inorganic Perovskites: Low-Cost Semiconductors with Intriguing Charge-Transport Properties. Nat. Rev. Mater..

[8] Yang Y., Hoang M.T., Bhardwaj A., Wilhelm M., Mathur S., Wang H. (2022). Perovskite solar cells based self-charging power packs: Fundamentals, applications and challenges. Nano Energy.

[9] Green M.A., Ho-Baillie A., Snaith H.J. (2014). The Emergence of Perovskite Solar Cells. Nat. Photonics.

[10] Seok S.I., Grätzel M., Park N.-G. (2018). Methodologies toward Highly Efficient Perovskite Solar Cells. Small.

[11] Möllmann A., Bialuschewski D., Fischer T., Tachibana Y., Mathur S., Guillon O. (2020). 6—Functional Metal Oxide Ceramics as Electron Transport Medium in Photovoltaics and Photo-Electrocatalysis. Elsevier Series on Advanced Ceramic Materials.

[12] Haque M.A., Sheikh A.D., Guan X., Wu T. (2017). Metal Oxides as Efficient Charge Transporters in Perovskite Solar Cells. Adv. Energy Mater..

[13] Singh T., Singh J., Miyasaka T. (2016). Role of Metal Oxide Electron-Transport Layer Modification on the Stability of High Performing Perovskite Solar Cells. ChemSusChem.

[14] Baena J.P.C., Steier L., Tress W., Saliba M., Neutzner S., Matsui T., Giordano F., Jacobsson T.J., Kandada A.R.S., Zakeeruddin S.M. (2015). Highly Efficient Planar Perovskite Solar Cells through Band Alignment Engineering. Energy Environ. Sci..

[15] Leijtens T., Eperon G.E., Pathak S., Abate A., Lee M.M., Snaith H.J. (2013). Overcoming Ultraviolet Light Instability of Sensitized TiO_2_ with Meso-Superstructured Organometal Tri-Halide Perovskite Solar Cells. Nat. Commun..

[16] Li W., Zhang W., Van Reenen S., Sutton R.J., Fan J., Haghighirad A.A., Johnston M.B., Wang L., Snaith H.J. (2016). Enhanced UV-light stability of planar heterojunction perovskite solar cells with caesium bromide interface modification. Energy Environ. Sci..

[17] Luo C.-W., Thilakan A.P., Li J.-X., Chen T.-P., Li S.-S., Chen C.-W., Yabushita A., Osada M., Tsukagoshi K., Sasaki T. (2020). UV degradation mechanism of TiO_2_-based perovskite solar cells studied by pump-probe spectroscopy. Photonics Sol. Energy Syst. VIII.

[18] Chaneliere C., Autran J.L., Devine R.A.B., Balland B. (1998). Tantalum Pentoxide (Ta_2_O_5_) Thin Films for Advanced Dielectric Applications. Mater. Sci. Eng. R. Rep..

[19] Hong Y., Fang Z., Yin B., Luo B., Zhao Y., Shi W., Li C. (2017). A Visible-Light-Driven Heterojunction for Enhanced Photocatalytic Water Splitting over Ta_2_O_5_ Modified g-C_3_N_4_ Photocatalyst. Int. J. Hydrogen Energy.

[20] Xu L., Guan J., Gao L., Sun Z. (2011). Preparation of Heterostructured Mesoporous In_2_O_3_/Ta_2_O_5_ Nanocomposites with Enhanced Photocatalytic Activity for Hydrogen Evolution. Catal. Commun..

[21] Xu L., Sun X., Tu H., Jia Q., Gong H., Guan J. (2016). Synchronous Etching-Epitaxial Growth Fabrication of Facet-Coupling NaTaO_3_/Ta_2_O_5_ Heterostructured Nanofibers for Enhanced Photocatalytic Hydrogen Production. Appl. Catal. B Environ..

[22] Cherevan A.S., Gebhardt P., Shearer C.J., Matsukawa M., Domen K., Eder D. (2014). Interface Engineering in Nanocarbon–Ta_2_O_5_ Hybrid Photocatalysts. Energy Environ. Sci..

[23] Zhou C., Shang L., Yu H., Bian T., Wu L.-Z., Tung C.-H., Zhang T. (2014). Mesoporous Plasmonic Au-Loaded Ta_2_O_5_ Nanocomposites for Efficient Visible Light Photocatalysis. Catal. Today.

[24] Jiang Y., Liu P., Liu Y., Liu X., Li F., Ni L., Yan Y., Huo P. (2016). Construction of Amorphous Ta_2_O_5_/g-C_3_N_4_ Nanosheet Hybrids with Superior Visible-Light Photoactivities for Organic Dye Degradation and Mechanism Insight. Sep. Purif. Technol..

[25] Zhu G., Lin T., Cui H., Zhao W., Zhang H., Huang F. (2016). Gray Ta_2_O_5_ Nanowires with Greatly Enhanced Photocatalytic Performance. ACS Appl. Mater. Interfaces.

[26] Cherevan A.S., Robbins S., Dieterle D., Gebhardt P., Wiesner U., Eder D. (2016). Ordered Gyroidal Tantalum Oxide Photocatalysts: Eliminating Diffusion Limitations and Tuning Surface Barriers. Nanoscale.

[27] Yu X., Li W., Li Z., Liu J., Hu P. (2017). Defect Engineered Ta_2_O_5_ Nanorod: One-Pot Synthesis, Visible-Light Driven Hydrogen Generation and Mechanism. Appl. Catal. B Environ..

[28] Yu X., Li W., Huang J., Li Z., Liu J., Hu P. (2018). Superstructure Ta_2_O_5_ Mesocrystals Derived from (NH_4_)_2_Ta_2_O_3_F_6_ Mesocrystals with Efficient Photocatalytic Activity. Dalton Trans..

[29] Cherevan A., Gebhardt P., Kunzmann A., Costa R.D., Eder D. (2018). Beware of Doping: Ta_2_O_5_ Nanotube Photocatalyst Using CNTs as Hard Templates. ACS Appl. Energy Mater..

[30] Yu X., Wei Y., Li Z., Liu J. (2017). One-Step Synthesis of the Single Crystal Ta_2_O_5_ Nanowires with Superior Hydrogen Production Activity. Mater. Lett..

[31] Tao C., Xu L., Guan J. (2013). Well-Dispersed Mesoporous Ta_2_O_5_ Submicrospheres: Enhanced Photocatalytic Activity by Tuning Heating Rate at Calcination. Chem. Eng. J..

[32] Sreethawong T., Ngamsinlapasathian S., Yoshikawa S. (2013). Facile Surfactant-Aided Sol–Gel Synthesis of Mesoporous-Assembled Ta_2_O_5_ Nanoparticles with Enhanced Photocatalytic H_2_ Production. J. Mol. Catal. A Chem..

[33] Lindmayer J., Allison J.F. (1975). Tantalum Pentoxide Anti-Reflective Coating. U.S. Patent.

[34] Lindmayer J. (1979). Tantalumoxide Antireflective Coating and Method of Forming Same. U.S. Patent.

[35] Rubio F., Dennis J., Albella J.M., Martinez-Duart J.M. (1983). Reactive Sputtered Ta_2_O_5_ Antireflection Coatings. Sol. Cells.

[36] Lü X., Ding S., Lin T., Mou X., Hong Z., Huang F. (2012). Ta_2_O_5_ Nanowires: A Novel Synthetic Method and Their Solar Energy Utilization. Dalton Trans..

[37] Jiang Q., Gao J., Yi L., Hu G., Zhang J. (2016). Enhanced Performance of Dye-Sensitized Solar Cells Based on P25/Ta_2_O_5_ Composite Films. Appl. Phys. A Mater. Sci. Process..

[38] Narangari P.R., Karuturi S.K., Wu Y., Wong-Leung J., Vora K., Lysevych M., Wan Y., Tan H.H., Jagadish C., Mokkapati S. (2019). Ultrathin Ta_2_O_5_ Electron-Selective Contacts for High Efficiency InP Solar Cells. Nanoscale.

[39] Lan J.-L., Cherng S.-J., Yang Y.-H., Zhang Q., Subramaniyan S., Ohuchi F.S., Jenekhe S.A., Cao G. (2014). The Effects of Ta_2_O_5_–ZnO Films as Cathodic Buffer Layers in Inverted Polymer Solar Cells. J. Mater. Chem. A.

[40] Ying Z., Chen W., Lin Y., He Z., Chen T., Zhu Y., Zhang X., Yang X., Djurišić A.B., He Z. (2019). Supersmooth Ta_2_O_5_/Ag/Polyetherimide Film as the Rear Transparent Electrode for High Performance Semitransparent Perovskite Solar Cells. Adv. Opt. Mater..

[41] Courtier N.E., Cave J.M., Foster J.M., Walker A.B., Richardson G. (2019). How transport layer properties affect perovskite solar cell performance: Insights from a coupled charge transport/ion migration model. Energy Environ. Sci..

[42] Awni R.A., Song Z., Chen C., Li C., Wang C., Razooqi M.A., Chen L., Wang X., Ellingson R.J., Li J.V. (2020). Influence of Charge Transport Layers on Capacitance Measured in Halide Perovskite Solar Cells. Joule.

[43] Bera A., Wu K., Sheikh A., Alarousu E., Mohammed O.F., Wu T. (2014). Perovskite Oxide SrTiO_3_ as an Efficient Electron Transporter for Hybrid Perovskite Solar Cells. J. Phys. Chem. C.

[44] Wang K., Olthof S., Subhani W.S., Jiang X., Cao Y., Duan L., Wang H., Du M., Liu S.F. (2020). Novel Inorganic Electron Transport Layers for Planar Perovskite Solar Cells: Progress and Prospective. Nano Energy.

[45] Chavan R.D., Parikh N., Tavakoli M.M., Prochowicz D., Kalam A., Bhoite P.H., Yadav P., Hong C.K. (2021). Band alignment and carrier recombination roles on the open circuit voltage of ETL-passivated perovskite photovoltaics. Int. J. Energy Res..

[46] Chavan R.D., Tavakoli M.M., Trivedi S., Prochowicz D., Kalam A., Yadav P., Bhoite P.H., Hong C.K. (2021). Interface Engineering of Mesoscopic Perovskite Solar Cells by Atomic Layer Deposition of Ta_2_O_5_. ACS Appl. Energy Mater..

[47] Möllmann A., Gedamu D., Vivo P., Frohnhoven R., Stadler D., Fischer T., Ka I., Steinhorst M., Nechache R., Rosei F. (2019). Highly Compact TiO_2_ Films by Spray Pyrolysis and Application in Perovskite Solar Cells. Adv. Eng. Mater..

[48] Brar L.K., Singla G., Pandey O.P. (2015). Evolution of Structural and Thermal Properties of Carbon-Coated TaC Nanopowder Synthesized by Single Step Reduction of Ta-Ethoxide. RSC Adv..

[49] Szymanowski H., Zabeida O., Klemberg-Sapieha J.E., Martinu L. (2005). Optical Properties and Microstructure of Plasma Deposited Ta_2_O_5_ and Nb_2_O_5_ Films. J. Vac. Sci. Technol. A.

[50] Sellers M.C.K., Seebauer E.G. (2011). Measurement Method for Carrier Concentration in TiO_2_ via the Mott–Schottky Approach. Thin Solid Films.

[51] Chun W.-J., Ishikawa A., Fujisawa H., Takata T., Kondo J.N., Hara M., Kawai M., Matsumoto Y., Domen K. (2003). Conduction and Valence Band Positions of Ta_2_O_5_, TaON, and Ta_3_N_5_ by UPS and Electrochemical Methods. J. Phys. Chem. B.

[52] Salado M., Kokal R.K., Calio L., Kazim S., Deepa M., Ahmad S. (2017). Identifying the Charge Generation Dynamics in Cs^+^-Based Triple Cation Mixed Perovskite Solar Cells. Phys. Chem. Chem. Phys..

[53] Krogmeier B., Staub F., Grabowski D., Rau U., Kirchartz T. (2018). Quantitative Analysis of the Transient Photoluminescence of CH_3_NH_3_PbI_3_/PC61BM Heterojunctions by Numerical Simulations. Sustain. Energy Fuels.

[54] Kulkarni A., Jena A., Chen H.-W., Sanehira Y., Ikegami M., Miyasaka T. (2016). Revealing and reducing the possible recombination loss within TiO_2_ compact layer by incorporating MgO layer in perovskite solar cells. Sol. Energy.

[55] Liu D., Yang J., Kelly T.L. (2014). Compact Layer Free Perovskite Solar Cells with 13.5% Efficiency. J. Am. Chem. Soc..

[56] Eperon G.E., Stranks S.D., Menelaou C., Johnston M.B., Herz L.M., Snaith H.J. (2014). Formamidinium Lead Trihalide: A Broadly Tunable Perovskite for Efficient Planar Heterojunction Solar Cells. Energy Environ. Sci..

[57] Kim Y.-H., Cho H., Heo J.H., Kim T.-S., Myoung N., Lee C.-L., Im S.H., Lee T.-W. (2015). Multicolored Organic/Inorganic Hybrid Perovskite Light-Emitting Diodes. Adv. Mater..

[58] Saliba M., Matsui T., Seo J.-Y., Domanski K., Correa-Baena J.-P., Nazeeruddin M.K., Zakeeruddin S.M., Tress W., Abate A., Hagfeldt A. (2016). Cesium-Containing Triple Cation Perovskite Solar Cells: Improved Stability, Reproducibility and High Efficiency. Energy Environ. Sci..

