# Supplementary material for: Tantalum Oxide as an Efficient Alternative Electron Transporting Layer for Perovskite Solar Cells
Source: Nanomaterials (Basel). 2022 Feb 25;12(5):780. doi: 10.3390/nano12050780 (PMC8912079; doi:10.3390/nano12050780)
Supplement: Supplementary file 1 [file nanomaterials-12-00780-s001.zip › nanomaterials-1597734-supplementary.pdf]

nanomaterials

Supporting Information (SI)

Tantalum Oxide as an Efficient Alternative Electron Transporting Layer for Perovskite Solar Cells

Meenal Deo [1,2,†], Alexander Möllmann [1,†], Jinane Haddad [3], Feray Ünlü [1], Ashish Kulkarni [3], Maning Liu [4], Yasuhiro Tachibana [4], Daniel Stadler [1], Aman Bhardwaj [1], Tim Ludwig [1], Thomas Kirchartz [3] and Sanjay Mathur [1,*]

[1] Institute of Inorganic Chemistry, University of Cologne, Greinstr. 6, 50939 Cologne, Germany; meenald@srmist.edu.in (M.D.); amoellmann@gmx.de (A.M.); feray.uenlue@uni-koeln.de (F.Ü.); daniel.stadler@nmwp.de (D.S.); aman.bhardwaj@uni-koeln.de (A.B.); tim.ludwig88@gmx.de (T.L.)
[2] Department of Physics and Nanotechnology, SRM Institute of Science and Technology, Kattankulathur, Chennai 603203, India
[3] IEK5-Photovoltaics, Forschungszentrum Jülich, 52425 Jülich, Germany; j.haddad@fz-juelich.de (J.H.); a.kulkarni@fz-juelich.de (A.K.); t.kirchartz@fz-juelich.de (T.K.)
[4] School of Engineering, RMIT University, Bundoora, VIC 3083, Australia; maning.liu@tuni.fi (M.L.); yasuhiro.tachibana@rmit.edu.au (Y.T.)
* Correspondence: sanjay.mathur@uni-koeln.de
† These authors contributed equally to this work.

S1: Procedure for the fabrication of perovskite solar cells:

Materials: bis(trifluoromethane)sulfonimide lithium salt (Li-TFSI, 99.95%), cesium iodide (99.999%), FTO/glass substrates (TEC 7, 2 mm), spiro-MeOTAD (99%, HPLC grade) and titanium diisopropoxide bis(acetylacetonate) (TDBA) 75 wt.% in isopropanol were purchased from Sigma-Aldrich. Furthermore, 30NRD TiO_2 nanoparticle paste and tris[2-(1H-pyrazol-1-yl)-4-tert-butylpyridine] cobalt(III) tri[bis-(trifluoromethane) sulfonimide] (FK209 Co(III), >98%) were purchased from Dyesol, Queanbeyan, Australia. PbI_2 (99.999%) and $PbBr_2$ (99.999%) were purchased from TCI Deutschland GmbH, Eschborn, Germany. Dimethyl sulfoxide (DMSO, extra dry, 99.7+%), N,N-dimethylformamide (DMF, extra dry, 99.8%), chlorobenzene (extra dry, 99.8%) and acetonitrile (99.9%) were bought from Acros Organics, Geel, Belgium. Absolute ethanol (HPLC grade) was purchased from Fisher Scientific, Massachusetts, United States. 4-Tert-butyilpyridine (4-TBP) was bought from Fluorochem. Formamidinium iodide (FAI) and methylammonium bromide (MABr) salts were synthesized according to recipes reported in the literature [1,2].

Device Fabrication: A defined part of each 2 cm × 2 cm FTO glass substrate was mechanically etched. These etched FTO substrates were then cleaned in an ultrasonic bath using Hellmanex III solution (0.5–2% in DI water), followed by DI water and absolute ethanol, for 20, 15 and 10 min, respectively. Before spin coating, the substrates were put into a UV–ozone chamber for 15 min to remove organic residuals from the surfaces and increase hydrophilicity.

The spray coating of TiO_2 was conducted on the cleaned FTO substrates with an Aldrich® chromatography sprayer (10 mL) at 450 °C using a 0.2 M titanium diisoproxide bisacetylacetonate (TDBA) diluted in absolute ethanol. After cooling down these films to 150 °C, a mesoporous TiO_2 layer was spin coated with an ethanolic suspension of TiO_2 nanoparticles (150 mg 30NRD TiO_2 paste in 1 mL EtOH) at 4000 rpm with 2000 rpm/s acceleration for 20 s, and the FTO contact area was covered with Kapton tape. The films were dried at 100 °C for 10 min and calcined at 450 °C for 30 min in air. The films were left to cool down to 150 °C and kept at this temperature until further use.

In a dry nitrogen glovebox, a perovskite solution was prepared following the recipe reported elsewhere [3] using a solution containing 0.95 M FAI, 1.1 M PbI_2, 0.19 M MABr, 0.2 M $PbBr_2$ and 0.06 M CsI in anhydrous DMF:DMSO 4:1 (v:v). Fifty microliters of this

solution was dropped onto the mesoporous TiO_2 layer and spin-coated at 1000 rpm with 500 rpm/s acceleration for 10 s and 6000 rpm with 2500 rpm/s acceleration for 20 s. Five seconds prior to the end, chlorobenzene (100 µL) was dripped in as an anti-solvent treatment. The perovskite thin films were annealed at 100 °C for 1 h. After annealing and cooling down to room temperature, a solution of spiro-OMeTAD was spin-coated at 4000 rpm with 2000 rpm/s acceleration for 45 s. The spiro-OMeTAD solution was prepared by dissolving 72.3 mg spiro-OMeTAD in 1 mL chlorobenzene. In this solution, 28.3 µL 4-TBP, 17.5 µL Li-TFSI solution (520 mg/mL in acetonitrile) and 29 µL FK209 (300 mg/mL in acetonitrile) were added and mixed with a vortex mixer.

After deposition, the solar cells were stored in a desiccator (relative humidity <10%) in the dark overnight. A gold layer 70 nm thick was thermally evaporated on the spiro-OMeTAD layer to form the back contact. The fabricated cells were stored in a desiccator and were left there for one further night prior to characterization.

For steady state photoluminescence and UV–Vis absorption measurements of the perovskite, the perovskite solution was spin coated as described above on glass, tantalum oxide coated glass and titanium oxide sprayed glass substrates.

Characterization of Devices: Current (J)–voltage (V) curves of the perovskite solar cells were measured with a 2420 Series SourceMeter (Keithley instruments) and a WACOM-WXS-140S-Super-L2 sun simulator with a two lamp Xenon/Halogen lamp system (class AAA) to simulate the AM 1.5G sunlight with incident power of 100 mW cm^{-2}. A contact box with metal mask with a thickness of 200 µm and an aperture area of 0.15 cm^2 was used for the device measurements. The devices were measured directly without preconditioning, and a black cardboard was used to protect the remaining cells from light soaking during the measurements. A sweep range between −0.2 and 1.2 V was used so that the V_{oc} was ~90% of the maximum applied potential. The scans were conducted with a sweep rate of 10 mV/sec in a forward, (−0.2 V to 1.2 V) and then in a reverse (1.2 V to −0.2 V) direction. For each stack, several cells were characterized to determine the photovoltaic parameters open-circuit potential (V_{oc}), fill factor (FF), short-circuit current (J_{sc}) and power conversion efficiency (PCE). A maximum power point (MPP) tracking measurement was conducted at a constant applied potential of 0.78 V. The external quantum efficiencies (EQE) were measured using a QEPVSI-b from Newport connected to a 150 W Xenon lamp and an AM1.5G optical filter. The chopper frequency was set to 30 Hz.

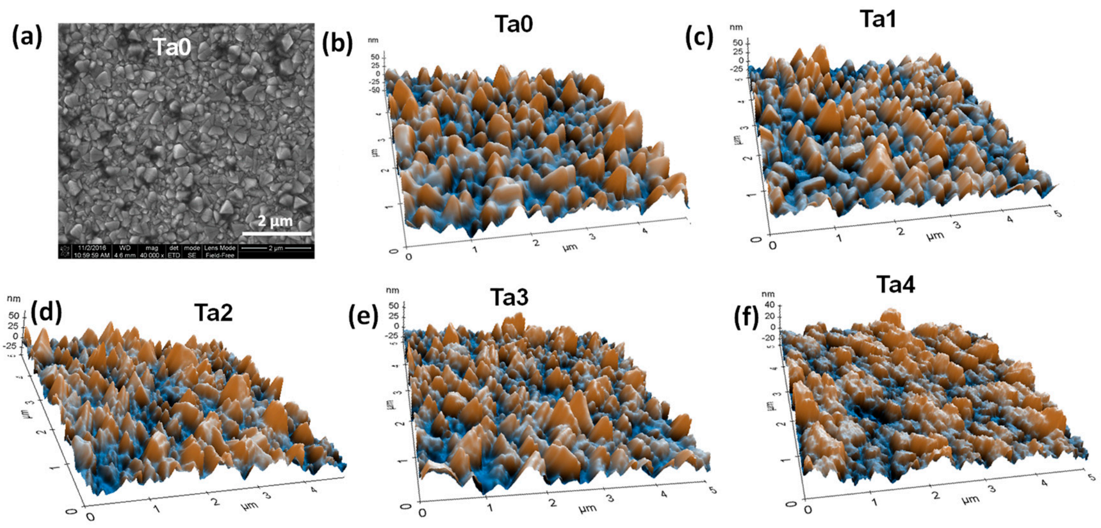

Figure S1. (**a**) Scanning electron microscopy (SEM) and (**b**) Atomic force microscopy (AFM) images of bare FTO, i.e., Ta0; AFM of the films (**c**) Ta1, (**d**) Ta2, (**e**) Ta3 and (**f**) Ta4.

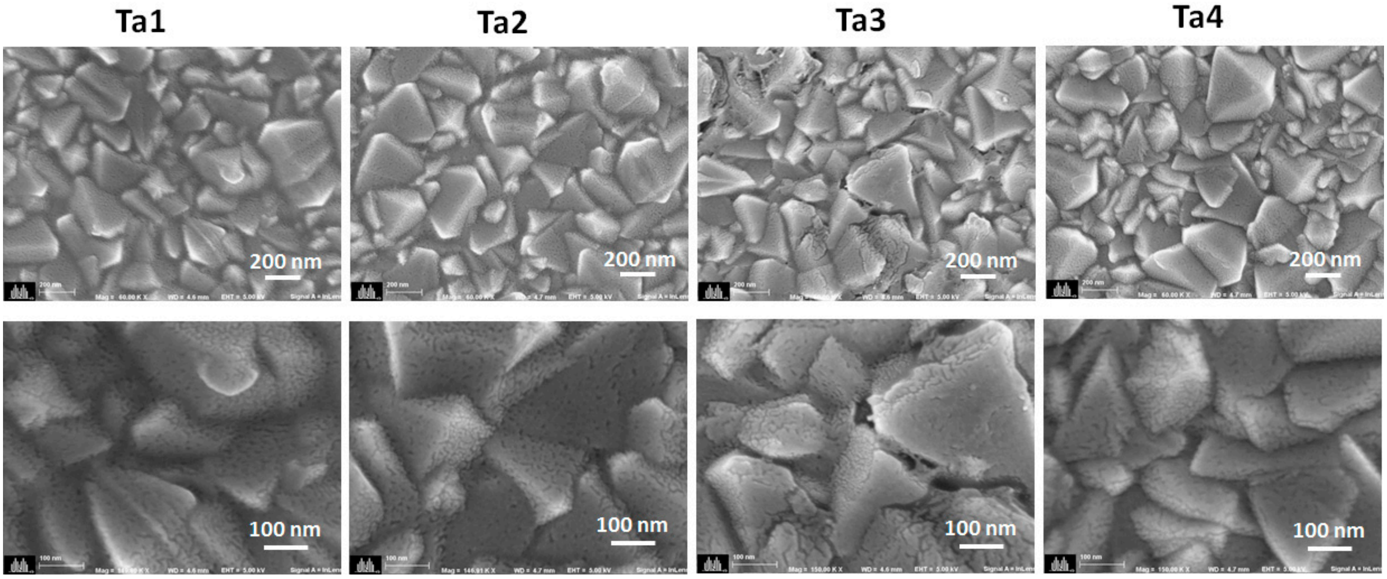

Figure S2. Morphologies of the films Ta1, Ta2, Ta3 and Ta4 by SEM with low and high magnifications.

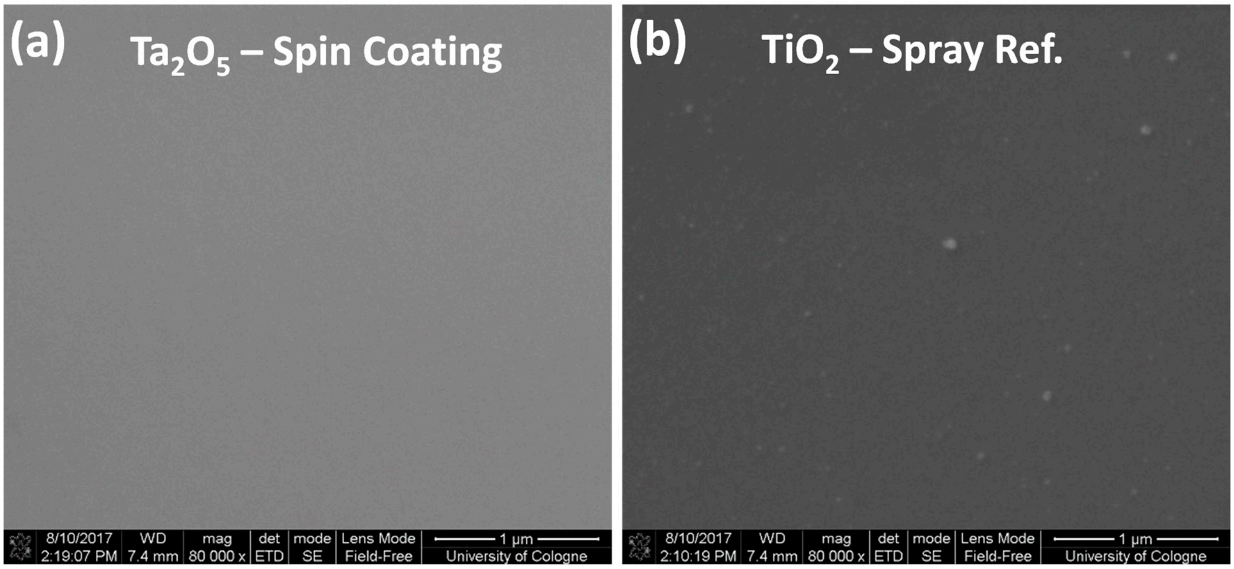

Figure S3. Surface morphologies of the films: (**a**) Ta_2O_5 by spin coating and (**b**) TiO_2 by spray pyrolysis prepared on silicon substrate.

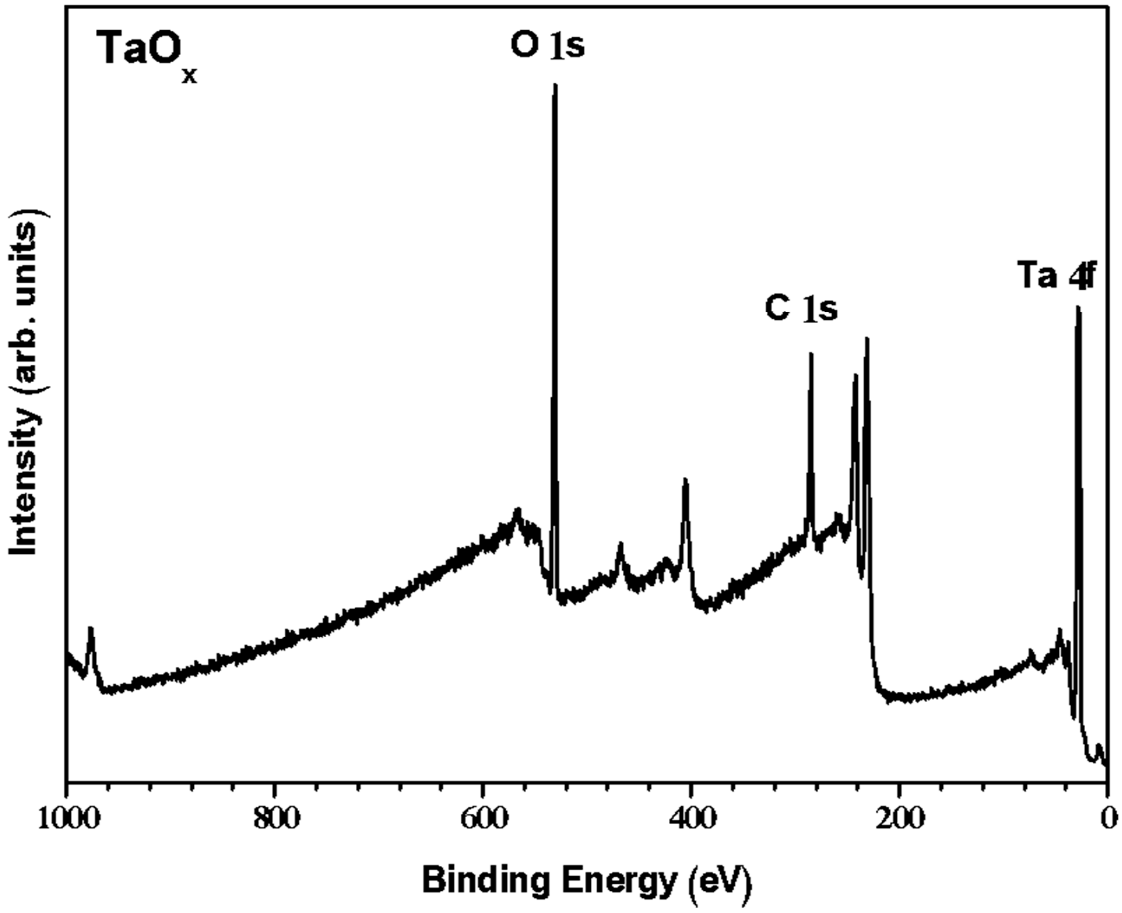

Figure S4. XPS survey spectrum of tantalum oxide thin film.

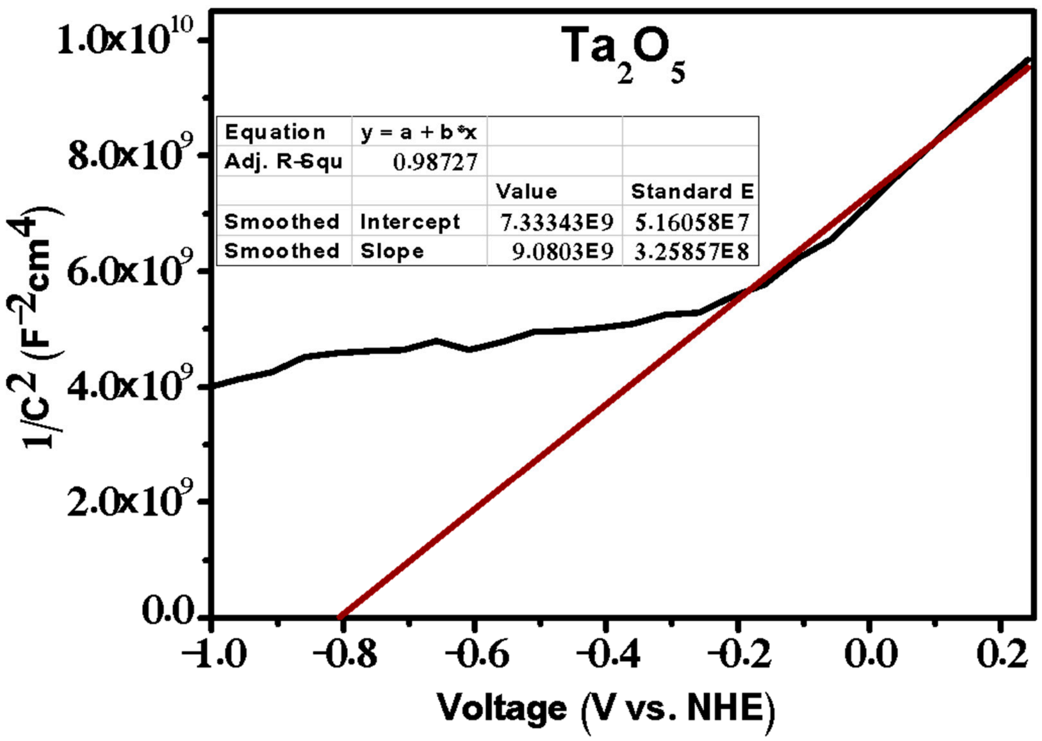

Figure S5. Mott–Schottky plot of Ta3 film dipped in 0.1 M Na_2SO_4 as an electrolyte recorded at 1 kHz.

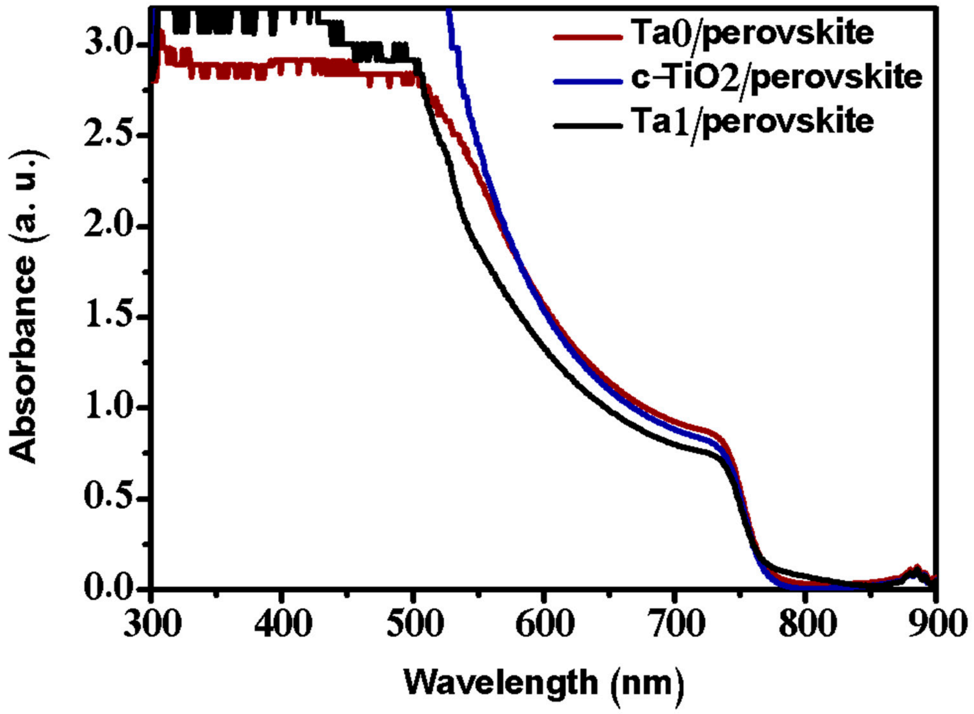

Figure S6. Absorption spectra of glass/perovskite, glass/c-TiO_2/perovskite and glass/Ta_2O_5/perovskite with ~9 nm Ta_2O_5 layer thickness (Ta1).

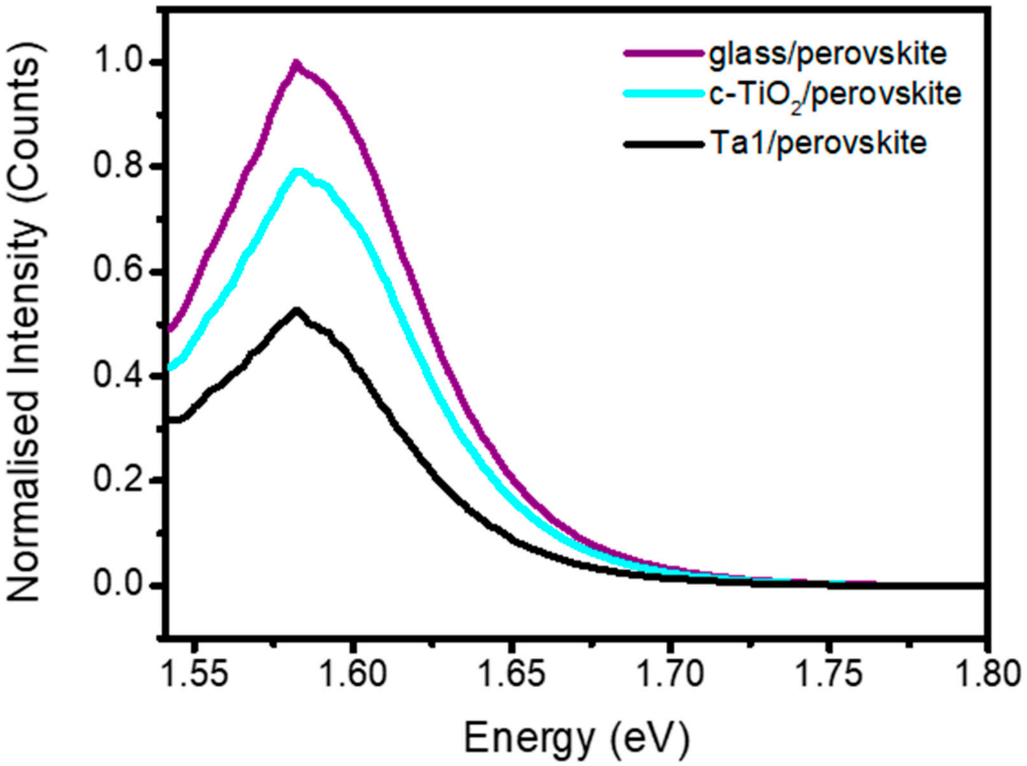

Figure S7. Normalized steady state photoluminescence spectra of glass/perovskite, glass/c-TiO_2/perovskite and glass/Ta_2O_5/perovskite with ~9 nm Ta_2O_5 layer (Ta1) at 625 nm excitation.

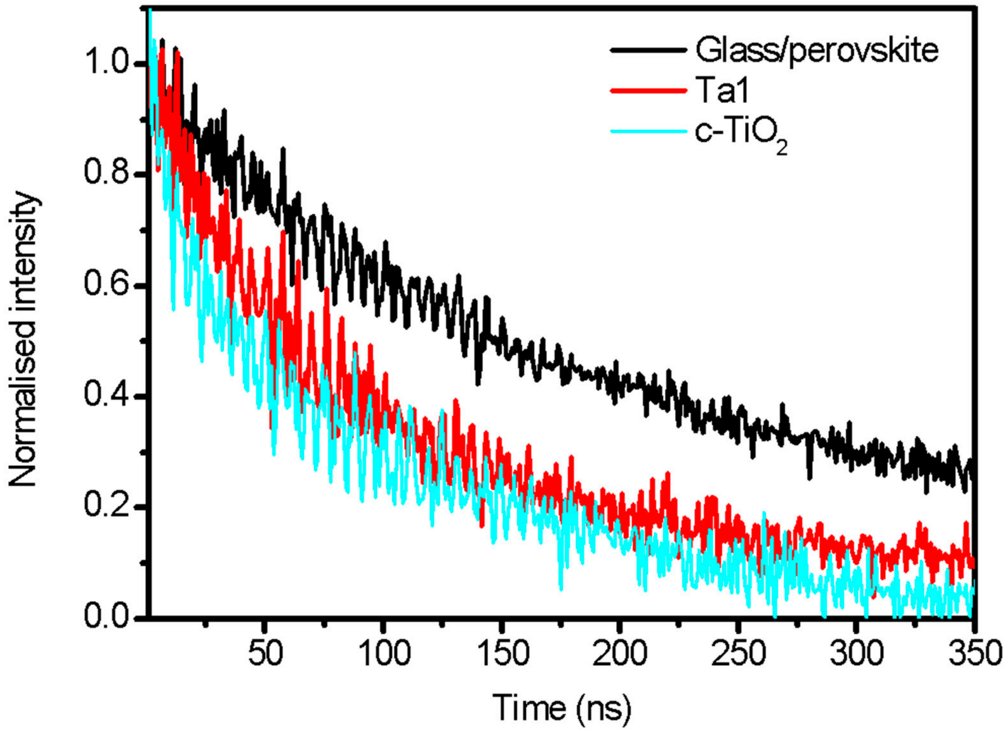

Figure S8. Transient photoluminescence spectra of glass/perovskite, glass/c-TiO_2/perovskite and glass/Ta_2O_5/perovskite with ~9 nm Ta_2O_5 layer (Ta1) at 625 nm excitation.

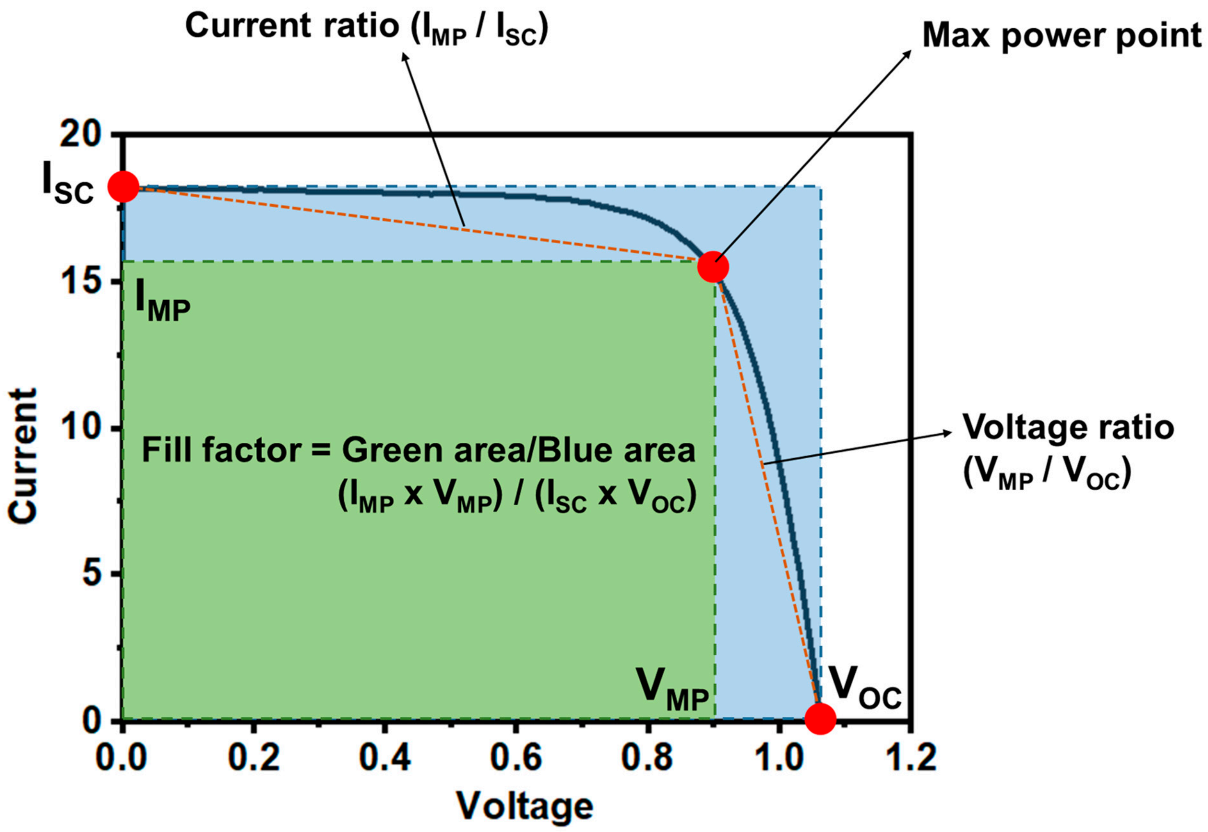

Figure S9. Illustration of the determination of fill factor for Ta1 champion device in reverse bias.

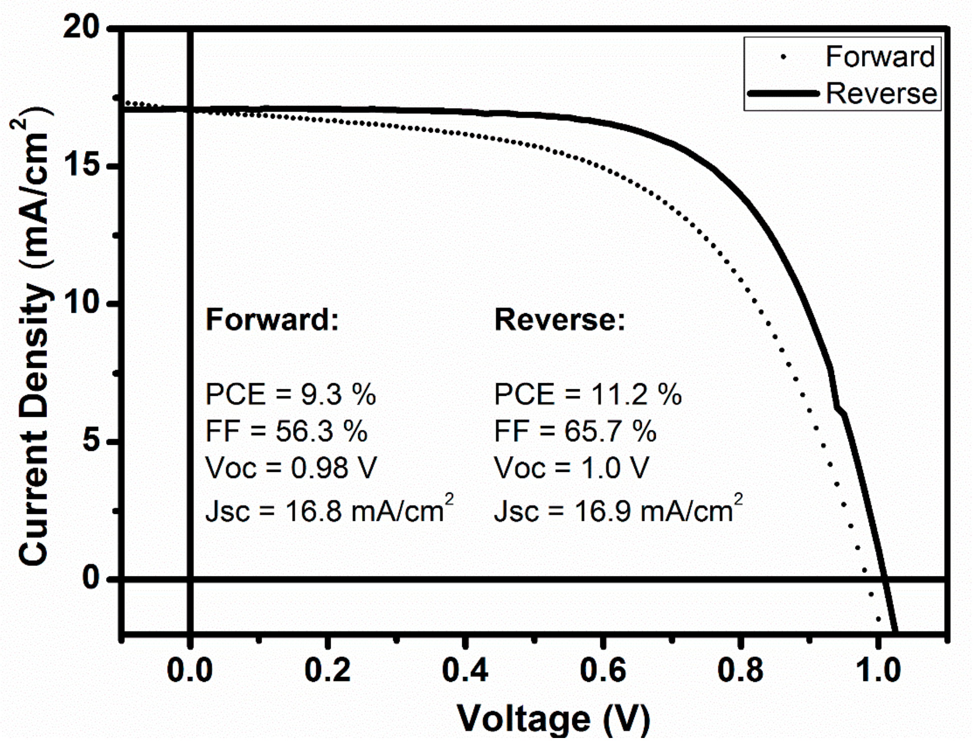

Figure S10. Reference cell J–V curves of perovskite solar cells employing compact and mesoporous TiO_2 as ETL.

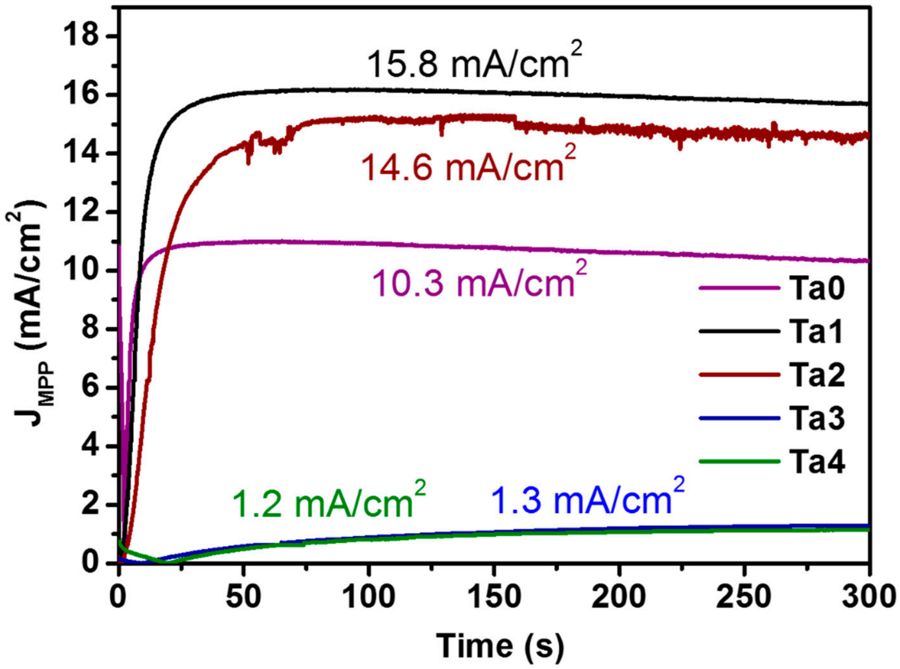

Figure S11. Measured photocurrent density of PSCs at maximum power (0.78 V applied bias) with varying tantalum oxide thickness.

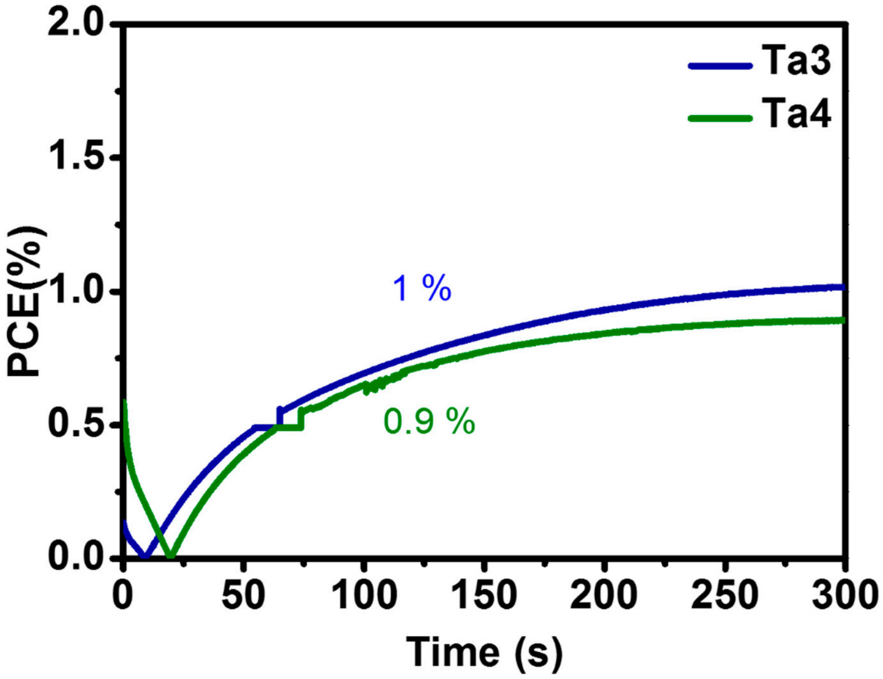

Figure S12. Calculated device efficiency of Ta3 and Ta4 PSCs from maximum power (0.78 V applied bias) tracking over 300 s.

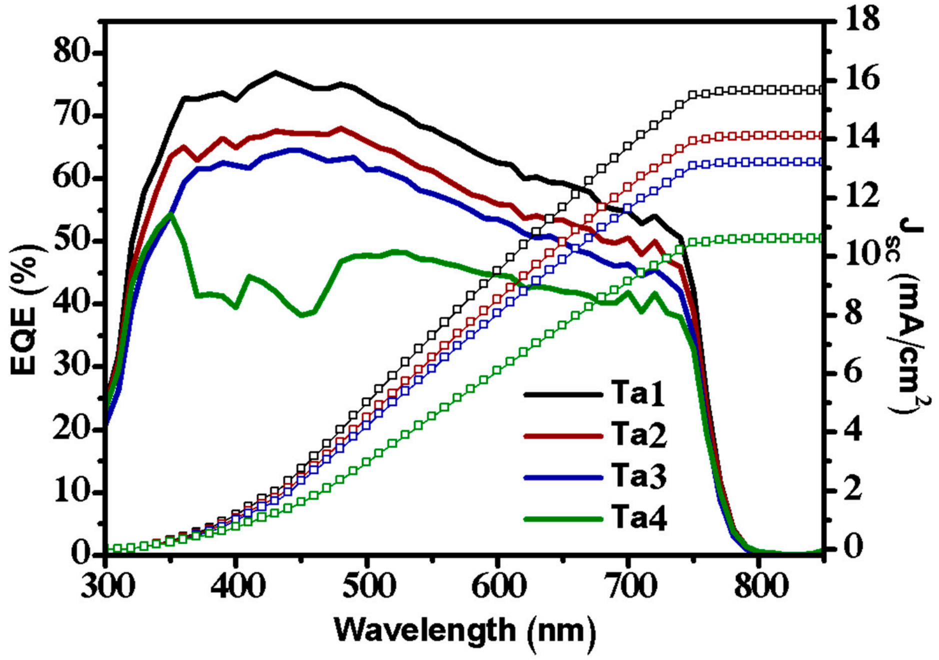

Figure S13. External quantum efficiency (EQE) spectra and corresponding integrated photocurrent densities of typical perovskite solar cells varying in Ta_2O_5 layer thickness.

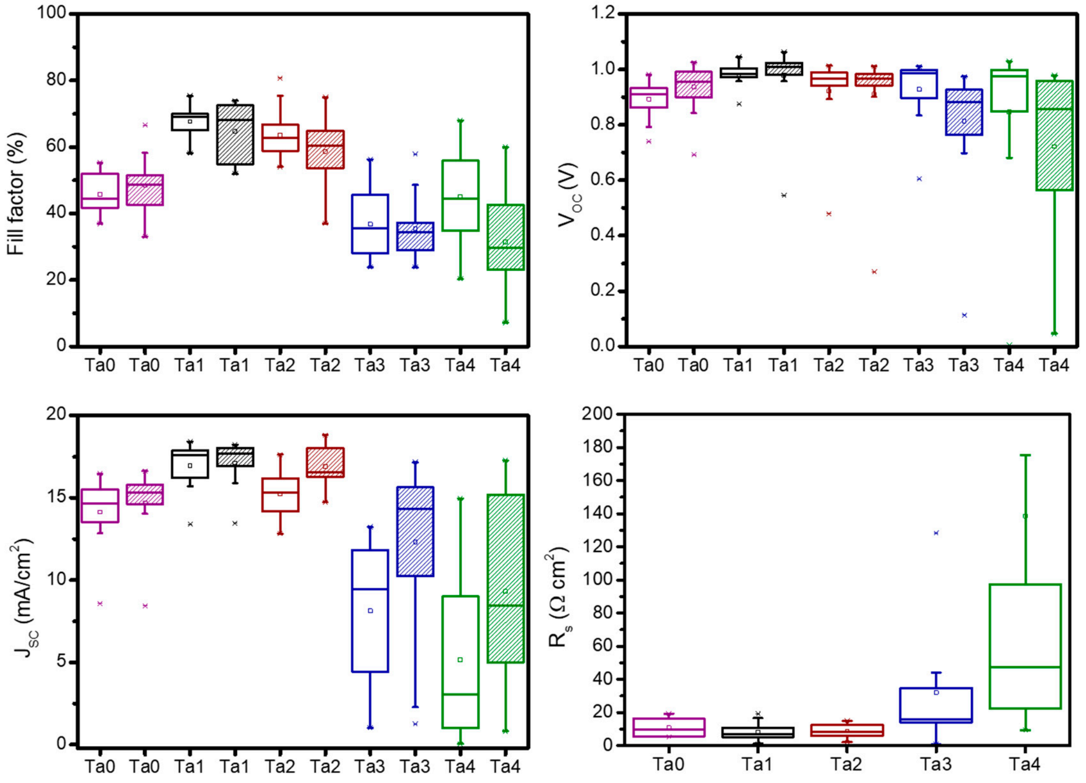

Figure S14. Box plot diagrams for the distribution of FF, V_{oc}, J_{sc} and R_s (only reverse sweep) extracted from the J–V curves.

References:

1. Eperon, G.E.; Stranks, S.D.; Menelaou, C.; Johnston, M.B.; Herz, L.M.; Snaith, H.J. Formamidinium Lead Trihalide: A Broadly Tunable Perovskite for Efficient Planar Heterojunction Solar Cells. *Energy Environ. Sci.* **2014**, *7*, 982–988.
2. Kim, Y.-H.; Cho, H.; Heo, J.H.; Kim, T.-S.; Myoung, N.; Lee, C.-L.; Im, S.H.; Lee, T.-W. Multicolored Organic/Inorganic Hybrid Perovskite Light-Emitting Diodes. *Adv. Mater*. **2015**, *27*, 1248–1254.
3. Saliba, M.; Matsui, T.; Seo, J.-Y.; Domanski, K.; Correa-Baena, J.-P.; Nazeeruddin, M.K.; Zakeeruddin, S.M.; Tress, W.; Abate, A.; Hagfeldt, A.; et al. Cesium-Containing Triple Cation Perovskite Solar Cells: Improved Stability, Reproducibility and High Efficiency. *Energy Environ. Sci.* **2016**, *9*, 1989.
